# Can the Spinal Cord Learn and Remember?

**DOI:** 10.1100/tsw.2008.106

**Published:** 2008-08-01

**Authors:** Pierre A. Guertin

**Affiliations:** ^1^ Neuroscience Unit, Laval University Medical Center (CHUL - CHUQ), Quebec City, Quebec, Canada; ^2^ Faculty of Medicine Department of Anatomy and Physiology, Laval University, Quebec City, Quebec, Canada

**Keywords:** spinal cord, plasticity, rehabilitation, pain, reflex

## Abstract

Learning and memory traditionally have been associated with cellular processes occurring in a specialized region of the brain called the hippocampus. However, recent data have provided strong evidence to suggest that comparable processes are also expressed in the spinal cord. Experiments performed mainly in spinal cord–transected animals have reported that, indeed, spinal-mediated functions, such as the stretch or flexion reflex, pain signaling, micturition, or locomotion, may undergo plasticity changes associated with partial functional recovery that occur spontaneously or conditionally. Many of the underlying cellular mechanisms strikingly resemble those found in the hippocampus. This mini-review reports, mainly, animal data that support the idea that other areas of the central nervous system, such as the spinal cord, can also learn and remember.

## INTRODUCTION

For many centuries, the spinal cord was considered to be simply a "relay" that carried information between the brain and organs, such as the skeletal muscle, the heart, or the skin. More recently, researchers have begun to understand that the spinal cord is, in fact, a complex structure that processes, transforms, and generates neuronal signals, and can undergo considerable plasticity.

The spinal cord has been found to control essential functions from simple motor reflexes to complex behavioral tasks including pain signaling, micturition, and locomotion. Many of the controlling spinal circuits have been found to undergo plasticity changes after distally located injuries. For instance, adult mice were shown to spontaneously re-express some involuntary hindlimb movements a few weeks after a complete, low-thoracic spinal transection[1,2,3]. Along this line, spinal cord–transected cats were found to recover the ability to step on a moving treadmill after several days of specialized training[4,5]. Against what has been generally believed, the spinal cord is apparently not a static and hard-wired system, but, instead, a complex neural structure capable of adaptation, learning, and memory like the rest of the brain. In fact, spinal cord plasticity appears to constitute an important component of motor skill acquisition in intact animals and humans in the course of normal life, and also occurs in response to neurological damage or disease.

## SIMPLE REFLEXES

One of the relatively simple reflexes involved in motor control is the spinal stretch reflex (SSR). It is known to be mediated by a monosynaptic pathway made of a primary afferent terminal (i.e., Ia afferent from a muscle spindle) and its postsynaptic target, the alpha-motoneuron located ventrally in the gray matter. Although simple and apparently static, the SSR has been found to adapt itself and possess its own memory (reviewed in [6,7]). For instance, SSR amplitude (tested using the H-reflex) was found to change in relation to diurnal rhythms[8]. Also, H-reflex amplitude can increase or decrease following reward-induced conditioning without changes in initial alpha-motoneuron tone[9]. Given that such conditioning was revealed to remain after a complete spinal cord transection, it has provided clear evidence of plasticity and memory processes in the normal spinal cord[10]. In fact, clear evidence suggests that such plasticity is mediated by changes in motoneuron physiological properties and synaptic terminals[11]. Early work from Wolpaw[6,7,8,9,10,11,12] uncovered the existence of learning and memory events in the spinal cord that were once believed to be present only in the brain. Clinically, this ability of the spinal cord to learn and change may be used to modulate spontaneous recovery[12] or exaggerated reflexes (spasticity) typically found after spinal cord injury (SCI)[13]. This is also supported by recent data obtained from normal individuals showing the long-term effects of a single cycling session on sustained H-reflex amplitude changes[14].

Another well-known spinal reflex involved in motor control is the flexion reflex. It has also been shown to be capable of learning and memory. For instance, the hindlimb flexion reflex elicited by paw stimulation in chronic decerebrate rats was found to produce marked and long-lasting alterations after tissue injury and local inflammation[15]. Such a phenomenon was shown to be mediated by a prolonged heterosynaptic facilitation of dorsal horn neurons[16]. Another very clear example of learning and memory in the spinal cord was found in spinal cord–transected rats that were given leg shocks whenever one hindlimb was extended. These animals learned to maintain a flexion response that decreased net shock exposure[17].

## PAIN SIGNALING

The most convincing signs of memory processes in the spinal cord are probably associated with experiments done in the field of chronic pain. Clear similarities have been found between some of the mechanisms underlying chronic pain in the spinal cord and memory in the hippocampus. For instance, long-term potentiation (LTP) and wind-up are both found in the spinal cord and the hippocampus. LTP is defined as the long-lasting enhancement in communication between two neurons that is found following their simultaneous stimulation and activation[18]. By enhancing communication between two neurons, LTP improves their ability to communicate with one another across a synapse. Because memories are believed to be stored at least in part within these synapses, LTP (together with long-term depression [or LTD]) is generally accepted as one of the key cellular mechanisms underlying learning and memory. First discovered in the hippocampus by Lomo and Andersen in 1966 (reviewed in [19]), it has since been found in other areas of the central nervous system, such as in the spinal cord, where it is often referred to as "central sensitization" (reviewed in [20]). Indeed, following intensive peripheral noxious stimuli or injury, increased synaptic efficacy occurs in sensory neurons of the dorsal horn. This "central sensitization" typically leads to a reduction in pain threshold, an amplification of pain responses, and allodynia, which increases the sensitivity of noninjured areas[21,22]. More specifically, central sensitization and the early phase of LTP described in the hippocampus have been found to share two main mechanisms: first, the phosphorylation of synaptic receptors; second, the insertion of new AMPA (alpha-amino-3-hydroxy-5-methyl-4-isoxazolepropionic acid) receptors into the postsynaptic membrane. However, there also are differences between the two phenomena. Hippocampal LTP is based only on synaptic strengthening, whereas central sensitization may also be associated with other mechanisms, such as changes in intrinsic membrane properties and/or neuronal networks (e.g., disinhibition). Regarding the late phase component, central sensitization and LTP are known to both lead to the transcription of immediate-early genes with CRE (cyclic recombinase) promoters[20]. As mentioned earlier, “wind-up” is another form of short-term plasticity that has been reported in the brain as well as in deep dorsal horn neurons during repeated electrical stimulation of pain fibers (C-fibers)[23]. The progressive postsynaptic depolarization triggered by such stimulation was found to be mediated by increased calcium influx through NMDA (N-methyl-D-aspartate) ionophores and voltage-dependent calcium channels, resulting in a facilitation of LTP induction[24,25].

## COMPLEX MOTOR TASKS

Several critical motor functions, such as respiration, micturition, and locomotion, are controlled and modulated by spinal cord neurons. For instance, regarding respiration, it is generally accepted that the brainstem neural network produces the main respiratory rhythm that is subsequently modulated by spinal cord neurons. Respiration is also recognized to be adaptable. For instance, there is a transient plasticity associated with both the onset and offset of a respiratory response to stimuli, such as hypoxia or hypercapnia. This transient change, called short-term potentiation (STP), refers to the progressive increase in respiratory activity in the first few cycles in response to hypoxia and to the slow decay back to baseline[26,27]**.** Another form of plasticity associated with respiration is called long-term facilitation (LTF). It is induced by intermittent hypoxia and was found to depend on serotonin release in the spinal cord[28]. This form of learning was shown to depend critically on BDNF (brain-derived neurotrophic factor) synthesis (but not NT-3) near spinal phrenic motoneurons[29].

Locomotion is another “adaptable” function largely controlled by spinal cord networks. The spinal cord has indeed been clearly shown to contain all the necessary circuits to generate the basic signals for walking. These circuits, which are localized for the most part in the lumbar spinal cord, have been called Central Pattern Generator (CPG) for locomotion by Grillner and Zangger[30]. We now know that after thoracic or cervical SCI, lumbar cord circuits such as those of the CPG do not die, but undergo substantial plasticity-related changes. Behavioral evidence of that has been reported recently in mice where spontaneous recovery represented by small amplitude nonlocomotor movements (or NLM) (i.e., nonbilaterally alternating movements) were detected 1 week after surgery in low-thoracic, completely spinal cord–transected (Tx) mice[1]. Moreover, small amplitude locomotor-like movements (or LM) (i.e., bilaterally alternating movements involving one or several joints) were found to be re-expressed 2 weeks after Tx[1,2,3]. Different levels of spontaneously occurring movements were reported in various strains (higher levels in CD1 than in C57BL/6 or BALB/C mice), showing that genetic backgrounds may influence plasticity events in the spinal cord[2]. Similar signs of spontaneous locomotor function recovery have also been shown in spinal cats[4], whereas comparable events have been reported in other spinal networks, such as the one that controls micturition (reviewed in [31]). Comparable signs of spontaneous recovery have also been reported in humans[32,33]. These observations in animal models strongly support the idea that spinal locomotor networks (i.e., CPG) undergo spontaneous adaptive changes after SCI that apparently aim at partially re-expressing some locomotor functions. Obviously, these changes do not suffice to restore normal functions, although results can be enhanced using specialized training methods. For instance, spinal cord–transected cats have been shown to undergo striking recovery levels using regular treadmill training with body weight support, drugs (i.e., primary afferent input modulators), and sensory stimulation[34]. Edgerton and colleagues also showed long-term memory events in completely spinal cord–transected cats that retained, for at least 6 weeks, the ability to step involuntarily on a treadmill generated “spontaneously” after several weeks of intensive training sessions[35]. Most recently, convincing data that demonstrated that the spinal cord (specifically CPG networks) can retain or remember learned processes were reported in Rossignol’s laboratory[5]. It was revealed that partially spinal-transected (T10) cats that learned to step almost normally on a moving treadmill after several days of specialized training (with sensory stimulation, but without drug) could indeed walk almost perfectly within hours after a subsequent complete transection (T13) of the cord. The detailed cellular processes underlying spontaneously or conditionally occurring functional recovery remain unclear. However, recent evidence suggests a role for immediate early genes, such as c-fos and nor-1, as well as for transmembrane receptors and neurotrophic factors (i.e., modulated expression levels, reviewed recently in [36]). Interestingly, as in the hippocampus, BDNF was reported recently to play a determinant role in learning; more specifically, in instrumental training-promoted re-expression of locomotor-like movements in complete paraplegic rats[37,38,39].

## CONCLUSION

Learning and memory processes in the spinal cord have not been studied as extensively as those in the hippocampus. Nonetheless, clear evidence of spinal plasticity associated with simple reflexes or more complex functions has been found recently. Similar mechanisms between learning and memory processes in the hippocampus and in the spinal cord have been reported. These findings strongly suggest that the spinal cord is an adaptable rather than a hard-wired system that, in turn, may encourage the development of innovative rehabilitation strategies to restore functions in SCI patients.
